# MDP, a database linking drug response data to genomic information, identifies dasatinib and statins as a combinatorial strategy to inhibit YAP/TAZ in cancer cells

**DOI:** 10.18632/oncotarget.5749

**Published:** 2015-10-15

**Authors:** Cristian Taccioli, Giovanni Sorrentino, Alessandro Zannini, Jimmy Caroli, Domenico Beneventano, Laura Anderlucci, Marco Lolli, Silvio Bicciato, Giannino Del Sal

**Affiliations:** ^1^ Department of Life Sciences, University of Modena and Reggio Emilia, Modena 41125, Italy; ^2^ Laboratorio Nazionale CIB (LNCIB), Area Science Park, Trieste 34149, Italy; ^3^ Dipartimento di Scienze della Vita, Università degli Studi di Trieste, Trieste 34149, Italy; ^4^ Dipartimento di Ingegneria “Enzo Ferrari”, Modena 41125, Italy; ^5^ Dipartimento di Scienze Statistiche, Bologna 40124, Italy; ^6^ Department of Science and Drug Technology, University of Torino, Torino 10125, Italy

**Keywords:** cancer, targeted therapy, Hippo pathway, small molecules, pharmacogenomics

## Abstract

Targeted anticancer therapies represent the most effective pharmacological strategies in terms of clinical responses. In this context, genetic alteration of several oncogenes represents an optimal predictor of response to targeted therapy. Integration of large-scale molecular and pharmacological data from cancer cell lines promises to be effective in the discovery of new genetic markers of drug sensitivity and of clinically relevant anticancer compounds. To define novel pharmacogenomic dependencies in cancer, we created the Mutations and Drugs Portal (MDP, http://mdp.unimore.it), a web accessible database that combines the cell-based NCI60 screening of more than 50,000 compounds with genomic data extracted from the Cancer Cell Line Encyclopedia and the NCI60 DTP projects. MDP can be queried for drugs active in cancer cell lines carrying mutations in specific cancer genes or for genetic markers associated to sensitivity or resistance to a given compound. As proof of performance, we interrogated MDP to identify both known and novel pharmacogenomics associations and unveiled an unpredicted combination of two FDA-approved compounds, namely statins and Dasatinib, as an effective strategy to potently inhibit YAP/TAZ in cancer cells.

## INTRODUCTION

Genetic alterations in cancer cells often generate cancer-specific dependencies that are exploited to develop targeted therapies. Mounting evidence shows that the number of cancer patients responding to targeted therapies has dramatically increased. For example, about half of melanomas show mutations in BRAF, making these cells sensitive to specific BRAF (Vemurafenib) and/or MEK inhibitors. Although the beneficial responses are not always durable [1], the likelihood of patients bearing this kind of mutations to respond to Vemurafenib or MEK inhibitors is high [2]. For those patients without these genetic alterations, unfortunately no dedicated therapies exist yet [3] and the identification of novel pharmacogenomics associations driven by large-scale genomic studies could undoubtedly provide information that may increase patients' chances to survive. However, the systematic identification of disease-specific genes associated to drug sensitivity or resistance is hampered by the lack of integrated large-scale genomic and pharmacological data. The NCI60 cancer cell line database [4] and the Cancer Cell Line Encyclopedia (CCLE) [5] are the most comprehensive large-scale information sets with multiple genomic and drug response resources. Due to the extensive pharmacological and genomic data available, these open source databases are prime candidates for integration and broad public access. Nevertheless, the lack of interconnection among these resources reduces the opportunities for molecular oncology and system pharmacology investigations. With the aim to develop novel therapies by identifying cancer cells with specific drug sensitivity as a result of genetic abnormalities, we designed the Mutations and Drugs Portal (MDP), a publicly accessible database that interconnects pharmacological information extracted from the NCI60 DTP screening with genomic data of the CCLE and NCI60 projects. Specifically, the MDP not only associates pharmacological and genomics data of the NCI60 repository but also correlates CCLE sequencing data (1,651 genes) to NCI60 drug response information (50,816 compound). Indeed, while NCI60 exome-sequencing of 15,000 genes can be linked to pharmacological data through Cell Miner [4], a web tool part of the NCI60 project, the high quality genomic variant information of the CCLE, focusing on a specific set of cancer related genes, still lacks of any integration with a large panel of drug response data. As compared to other tools, whose pharmacological database is limited to few hundreds compounds [6, 7], MDP integration of CCLE sequencing and NCI60 drug data leverages the possibility to identify candidate compounds or genomic markers of response from cancer cell lines models. In this study, querying MDP for drugs active on cells bearing inactivating mutation in NF2 (Neurofibromatosis 2/Merlin) gene, a condition associated with hyperactivation of the oncogenes YAP and TAZ, we identified statins and Dasatinib as a pharmacological combination able to potently inhibit YAP/TAZ function in cancer cells.

## RESULTS

### Database content and architecture

**Figure 1:**
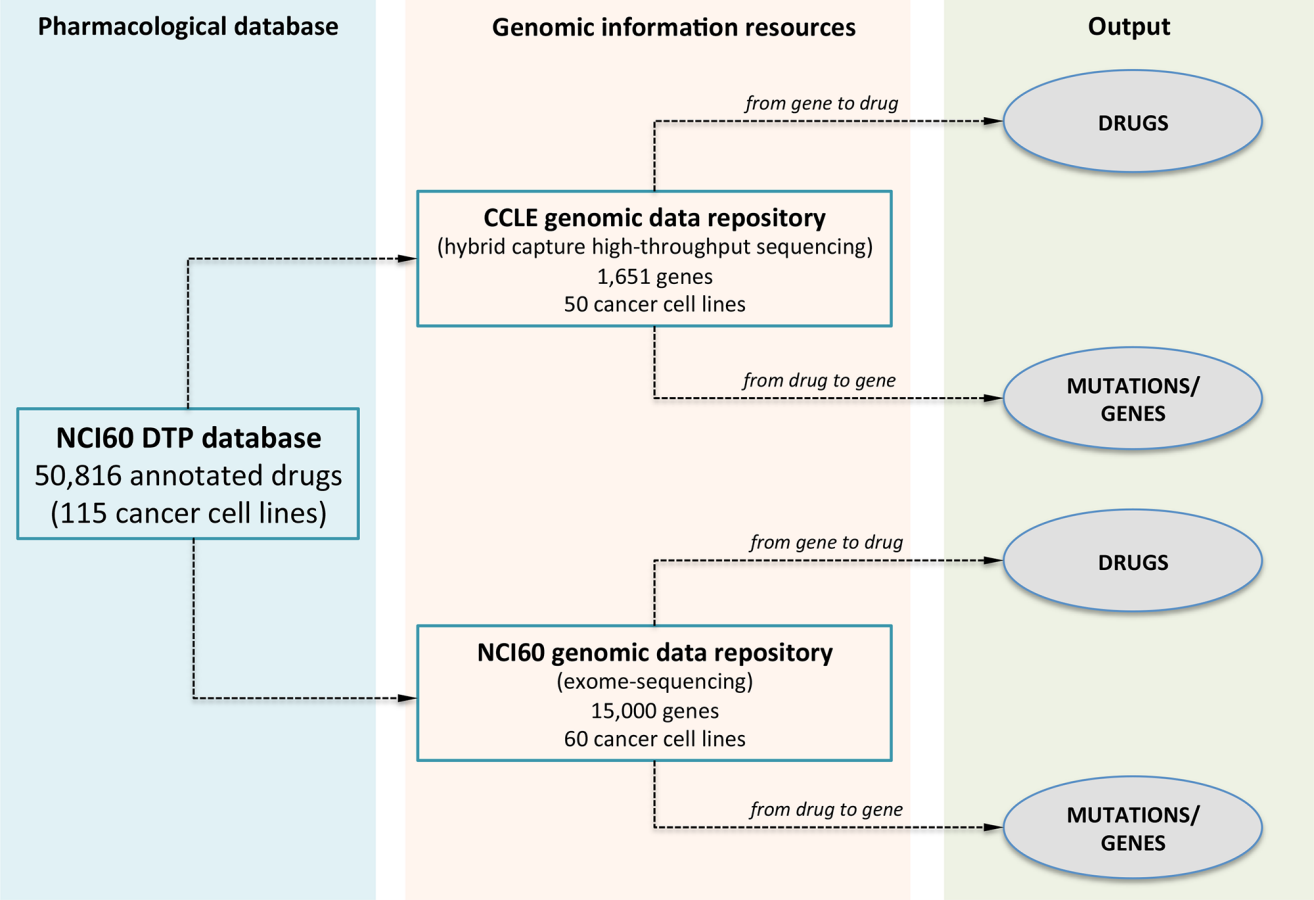
The database scheme of MDP MDP allows identifying compounds with statistically significant anticancer activity (*from gene to drug*) or the most enriched mutations for a selected compound (*from drug to gene*). Pharmacological data are from the NCI60 screening of 50,816 compounds on 115 cancer cell lines; genomic information is from the CCLE and NCI60 databases and comprises the high-throughput screening of 1,651 cancer-related genes on 50 cancer cell lines (CCLE) and of 15, 000 genes on 60 cell lines (NCI60). MDP can be interrogated to retrieve the most significant anticancer molecules selecting a gene or mutation using the *from gene to drug* analysis or to search for the most enriched mutation given a specific compound using the *from drug to gene query*.

The MDP database builds on exome-sequencing and drug response data from the NCI60 and the CCLE datasets. The National Cancer Institute (NCI) anticancer drug screen was originally developed to identify compounds with growth-inhibitory or toxic effects on a panel of 60 human tumour cell lines (NCI60), representing different tumour types [8, 9]. The aim of the project was to prioritize compounds for their ability to inhibit, *in vitro*, growth of human cancer cell lines from tissues as breast, prostate, lung, colon, ovary, kidney, central nervous system and from melanomas and leukemias. In the NCI60 screen, drug activity is measured in terms of GI_50_, the log of compound concentration required to cause 50% of growth inhibition whereas molecular data of cell lines includes DNA copy number, single nucleotide polymorphisms (SNPs) [10, 11], whole-exome sequencing [12], and RNA/micro-RNA expression profiles [13–15]. In particular, exome-sequencing data are available for 15,000 genes. Nowadays, the database of the NCI60 Developmental Therapeutics Program (NCI60 DTP) is by far the largest public repository of pharmacological and genomics data comprising response characteristics of cancer cell lines to more than 50,000 compounds. The Broad-Novartis Cancer Cell Line Encyclopedia (CCLE) instead, contains genome-wide DNA copy number, gene expression profiles for 960 human cancer cell lines, drug response information for 24 anticancer drugs across 504 cell lines, and the mutational status of 1,651 genes determined by hybrid capture high-throughput sequencing [5]. In essence, the NCI60 provides the largest variety of compounds and a comprehensive profiling of exonic variants, while CCLE offers the complete panel of cell lines, a profound sequencing depth, although limited to slightly more than 1,600 cancer-related genes, but rather limited pharmacological information. Given these premises, the aim of MDP is bridging NCI60 uniquely large number of drug sensitivity data with both CCLE and NCI60 DTP gene sequencing information. As such, MDP can query drug sensitivity data for 50,816 compounds on CCLE and NCI60 sequenced cell lines (see Figure 1). The guiding element, used to link NCI60 pharmacological information to CCLE and NCI60 genomic data, is the set of common cancer cell lines between the NCI60 drug screening (now available for 115 cell lines) and the CCLE and NCI60 genomic repositories, comprising 50 and 60 cell lines, respectively. Using these common cell lines, MDP can query NCI60 pharmacological data and CCLE and NCI-60 genomic information to identify drugs correlated to gene mutations (*from gene to drug*) or gene mutations associated to drug response (*from drug to gene*).

### Web interface

The web interface allows querying associations between genomic and pharmacological data in either direction depending on the particular question of interest. In the *Analysis* page, selecting the *from gene to drug* section, it is possible to start from a gene to retrieve compounds or drug families with growth-inhibitory effects on cancer cell lines carrying mutations on that gene. Instead, using the *from drug to gene* analysis, the user can input a drug to identify which genomic mutations affect the sensitivity of the cancer cell lines. Both types of queries can be performed selecting the genomic information of the 1,651 oncogenes from CCLE (*run analysis on CCLE*) or the whole set of 15,000 human genes from NCI60 repository (*run analysis on NCI60*).

**Figure 2:**
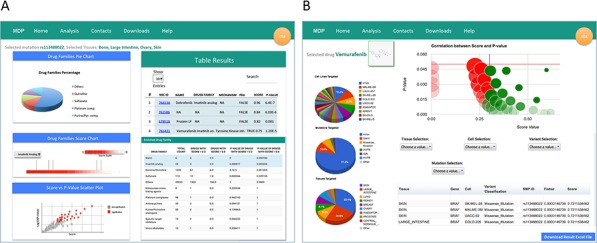
The result pages MDP mutation result pages contain different information depending on the type of query. **A.** When performing a *from gene to drug* analysis, the result page reports a list of molecules that show a statistically significant activity on cell lines bearing mutations on selected gene/s. In particular, the result page contains the *Table Results*, the *Enriched Drug Families* table and three plots charting the drug families frequency distribution of the complete pool of significant drugs, the score of each drug belonging to a particular drug family, and the relationship between the score and the *p*-value for each drug. **B.** When using the *from drug to gene* analysis, MDP returns a *Volcano Plot* highlighting gene variants significantly associated (*p*-value < 0.05 and score > 0.3) to sensitivity (green bubbles) or resistance (red bubbles). The size of the bubbles is proportional to the total number of mutations in sensitive/resistant cancer cell lines. Table and plots summarize the characteristics and distributions of gene variants associated to sensitivity or resistance

Specifically, when selecting the *from gene to drug* section, both *run analysis on CCLE* and *run analysis on NCI60* buttons re-direct to a web page where the user can query for the gene names or the SNP Ids of interest [16]. Multiple gene names or dbSNP Ids can be inputted at the same time to explore drug sensitivity to combinations of multiple genes or SNPs. Once selected the genomic entity, the query can be limited to subsets of tissues (i.e., cell lines) and of variant classifications (i.e., mutation types). Available gene names, dbSNP Ids, and mutation types will vary depending on the selected sequencing data, meaning that the search will be less flexible when selecting the high-depth genomic sequencing of the 1,651 cancer-related genes from CCLE. The output of *from gene to drug* analysis is a list of molecules that show a statistically significant activity on cell lines bearing specific mutations on the selected gene/s (Figure 2A). Results are returned using graphical and interactive representations and the list of molecules can be downloaded for post-processing evaluations. In particular, the result page contains two main tables, i.e., the *Table Results* and the *Enriched Drug Families* table. The *Table Results* lists the compound NSC ID, the compound name, the drug family, mechanism of action and FDA status, and the enrichment score and *p*-value, and can be sorted, ordered and searched to select specific characteristics of the outputted molecules. Instead, the *Enriched Drug Families* table shows the total sensitivity enrichment of each drug family when compared to the total number of compounds. Drug families are ranked according to the *p*-value of drugs with an enrichment score higher than 0.6. Furthermore, the result page contains three different plots:

1. a pie-chart showing the drug families frequency distribution of the complete pool of significant drugs;
2. a barplot showing the score of each drug belonging to a particular drug family;
3. a scatterplot showing the relationship between the score and the *p*-value for each drug and highlighting in red statistically significant compounds for a specific drug family.

When using the *from drug to gene* analysis, MDP identifies genomic variants that might be related to the sensitivity or resistance of cancer cell lines to a specific compound. The overall output is summarized in the *Volcano Plot* highlighting gene variants significantly associated to sensitivity (green bubbles) or resistance (red bubbles) to the selected molecule (*p*-value < 0.05 and score > 0.3). The size of the bubbles is proportional to the total number of mutations found in cancer cell lines (Figure 2B). The detail output lists, for gene variants associated to sensitivity or resistance, the tissue type, the gene carrying the mutation, the affected cell line, the variant classification, the mutation type, the chromosomal localization, the SNP ID, the enrichment score and *p*-value. The detailed results also include three pie charts showing the frequency of the identified variants in each cell line (*Cell Lines Analysis Distribution*), in each tissue (*Tissues Analysis Distribution*), and for each variant classification (*Variant Type Analysis Distribution*).

### Validation of known dependencies

To determine whether MDP could be used to find out real pharmacogenomic associations between somatic gene mutations and sensitivity to specific drugs, we decided to interrogate MDP for genes on which targeted therapies have been already established and clinically proved. In particular, the Mitogen-activated protein kinase (MAPK) pathway is involved in the control of proliferation of both normal and transformed cells [17]. MAPK cascade and its downstream kinase MEK (Mitogen-activated protein kinase) are activated by the Serine/Threonine Kinase RAF [17]. Somatic BRAF mutations, in particular the V600E mutation within the kinase domain, have been frequently found in melanomas, thyroid, colorectal, and lung cancer [18]. For these reasons, the oncogenic RAF-MAPK axis is nowadays used as a target for cancer therapies and specific BRAF and MEK inhibitors have been now approved by FDA (US Food and Drug Administration) for the treatment of diseases such as melanoma and lung cancer [19, 20].

**Figure 3:**
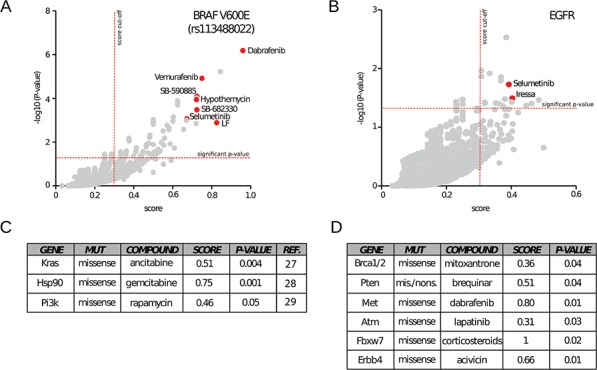
Application of MDP to investigate known dependencies MDP has been tested for its ability to retrieve known dependencies between gene variants and drugs. **A.** When searching for drugs active on cell lines bearing the V600E BRAF mutation (SNP Id rs113488022), MDP identifies well-known inhibitors such as Dabrafenib, Selumetinib and Vemurafenib. The scatter plot highlights the significance thresholds in terms of scores (vertical line, cut off of 0.3) and *p*-values (horizontal line, cut off of 0.05). **B.** The same graphical representation highlights Selumetinib and Iressa as effective drugs on cells with mutations on EGFR. **C.** Drugs statistically relevant on cancer cell lines with mutations of Kras, Hsp90 or Pi3K. **D.** Drugs statistically relevant on cancer cell lines with mutations of Brca1 and 2, Pten, Met, Atm, Fbxw7, and Erbb4 genes.

Thus, we interrogated MDP searching for drugs with selective cytotoxicity for cancer cells bearing BRAF mutations. Since the V600E accounts for the majority of BRAF mutations in cancer, we used its specific dbSNP Id number (rs113488022) to query CCLE genomic data. When considering all tissues, the analysis identified, out of 5,793 drugs, 197 drugs with significant selectivity for the BRAF-mutated cell lines. Interestingly, among the top 20 score-ranked molecules we identified seven compounds that are already associated with BRAF mutations. In particular, we identified three BRAF inhibitors: Dabrafenib [19] ( *p* = 6.48 × 10^−7^), Vemurafenib [20] ( *p* = 1.2 × 10^−5^) and SB-590885-AAD [21] ( *p* = 7.96 × 10^−5^); three MEK inhibitors: Hypothemycin [22] ( *p* = 9.49 × 10^−5^), Selumetinib [23] ( *p* = 0.0008) and protein LF (anthrax lethal factor, *p* = 0.001) [24]; and a tyrosine kinase inhibitor: SB-682330-A ( *p* = 7.96 × 10^−5^) (Figure 3A). Similar results were obtained querying MDP with NCI60 genomic data. Notably, Vemurafenib and Dabrafenib are FDA-approved and currently used for the treatment of melanoma, whereas Selumetinib is under clinical trials [25]. These results demonstrate the capacity of MDP to identify drugs with exquisite selectivity for specific mutations. It is of major interest to note that all the other molecules identified in our analysis could potentially act as BRAF-MEK inhibitors perhaps with better therapeutic activity and/or pharmacodynamic properties. As an example, the compound NSC-682449 (Benzo[1,2-b:4,5-b']dithiophene-4,8-dione, 2-(1-hydroxyethyl)), which falls within the 10 top ranked drugs, is surely worth of further investigations.

Next, we searched for pharmacogenomic associations with mutations in one of the most clinically relevant tyrosine kinase receptor: the Epidermal Growth Factor Receptor (EGFR). Mutations that lead to EGFR overexpression or hyperactivity have been associated with a number of cancers, including lung cancer [25]. To further demonstrate the reliability of MDP, we found a statistical significant association between the EGFR inhibitor Gefitinib (Iressa) (*p*-value = 0.03) [26] and the tyrosine kinase inhibitor Selumetinib (*p*-value = 0.03) with somatic missense EGFR mutations in CCLE cancer cell lines (Figure 3B).

We also successfully identified a number of other known pharmacogenomic associations such as: cytidine derivatives with KRAS mutations [27], gemcitabine with HSP90 mutations [28] and PI3K/AKT/mTOR inhibitors Sirolimus (Rapamycin) with PI3K missense mutations [29] (Figure 3C). Altogether, these results demonstrate that MDP resource can easily identify already known pharmacogenomic associations and might be a conceivable starting point to unveil and design new rational targeted therapies.

### Identification of new dependencies

Several other new pharmacogenomic associations that we identified cannot be explained with our current biological knowledge but surely generate novel interesting hypothesis to be validated with *in vitro* experiments. For example MDP suggests that BRCA1 and BRCA2 missense mutations are associated with sensitivity to Mitoxantrone; PTEN mutations with sensitivity to Brequinar; MET mutations with sensitivity to Dabrafenib; ATM mutations with sensitivity to Lapatinib; FBXW7 mutations with sensitivity to Corticosteroids (Prednisolone and Fluorometholone) and ERBB4 mutations with sensitivity to Acivicin (Figure 3D). If validated, all these associations could open new therapeutic opportunities for several tumours lacking approved targeted therapies.

Having tested its robustness and reliability, we used MDP to identify those compounds that might be able to inhibit proliferation of cancer cells with aberrant nuclear YAP/TAZ activation. YAP/TAZ are transcriptional cofactors that, in tumours, are deregulated by loss of cell polarity and EMT (epithelial-to-mesenchymal transition) and are highly expressed in cells with cancer stem cells properties and metastatic potential [30]. Increased YAP/TAZ transcriptional activity is frequent in several human malignancies, making YAP/TAZ an ideal therapeutic target for cancer. The main genetic lesions associated with YAP and TAZ hyperactivation, excluding the occasional gene amplification of chromosome locus 11q22 [31], are represented by mutations occurring on the genes encoding for members of the Hippo signalling pathway. Indeed, the Hippo pathway is the major negative regulator of YAP/TAZ transcriptional activity and, as such, is a potent regulator of cellular proliferation, differentiation, and tissue homeostasis [38]. Among the members of the Hippo pathway, mutations in the gene NF2 are the most frequent alterations [32]. Germline mutations in the NF2 gene is the cause of the Neurofibromatosis type 2, a tumour-prone disorder characterized by the development of multiple schwannomas and meningiomas, but importantly, NF2 is also frequently inactivated in human malignant pleural mesothelioma, a disease where YAP is frequently activated [33].

Based on these premises, we used MDP to seek for compounds that could specifically target cancer cells bearing NF2 gene mutations and thus with aberrant YAP/TAZ activity. In particular, we queried the association of NF2 missense and non-sense mutations and drugs (*from gene to drug*) on all CCLE cell lines and identified the most enriched family drugs. Interestingly, the Enriched Drug Families table of this query highlighted Statins (*p*-value < 0.01) and Imatinib analogues (*p*-value < 0.01) as the only two classes of drugs significantly associated to drug sensitivity in cancer cell lines harbouring NF2 mutations (Supplementary Figure 1). Of note, these classes of drugs were identified as statistically significant also by querying MDP with NCI60 data.

**Figure 4:**
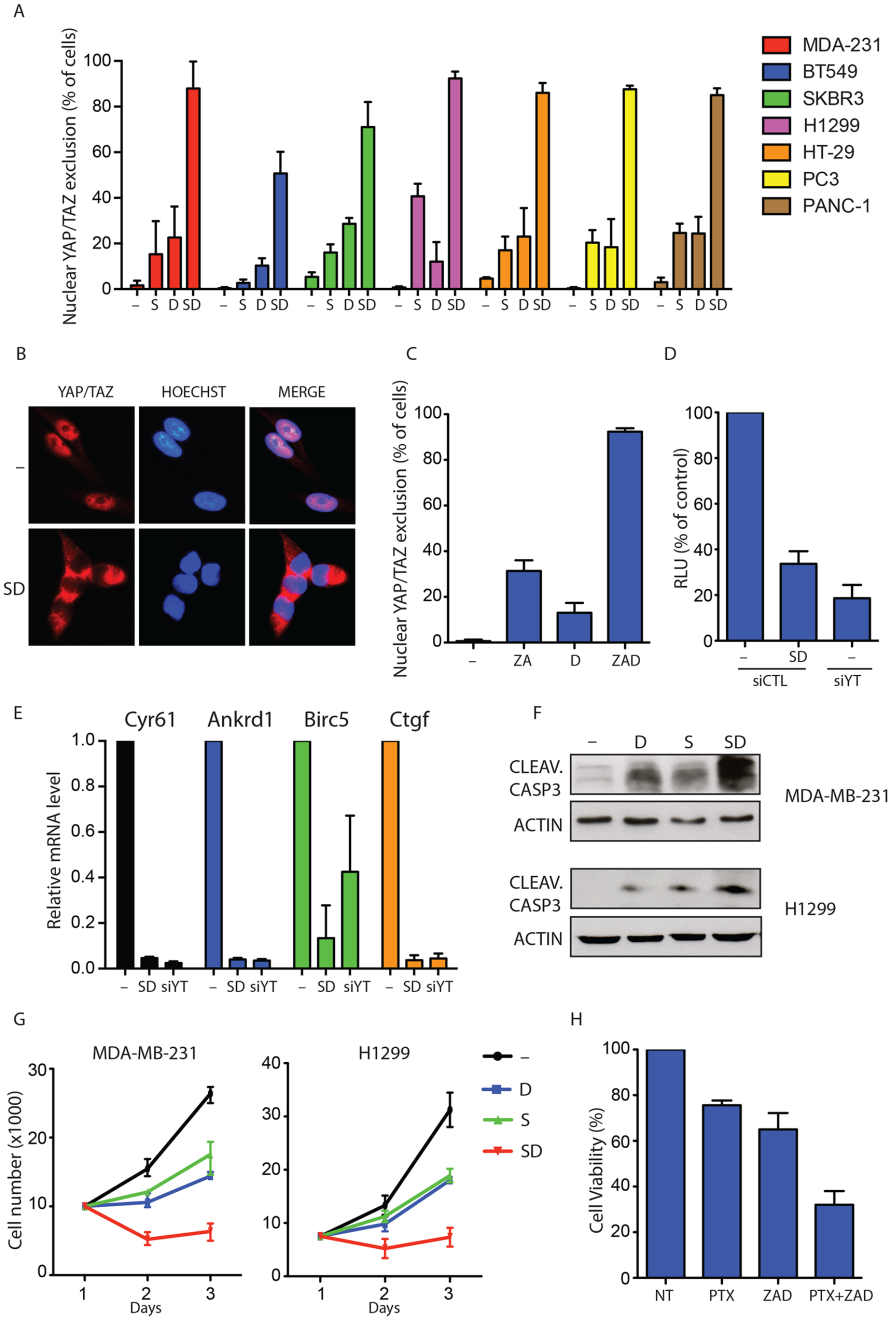
Dasatinib and statin combination fully blunts YAP/TAZ nuclear activity **A.** Quantification of cells with complete YAP/TAZ nuclear exclusion in seven cancer cell lines treated with vehicle (−), statin 1 μM (S) and Dasatinib 1 μM (D) alone or in combination (SD) for 24 h. Data are derived from *n* = 3 independent experiments where at least 300 cells were scored. Error bars represent mean ± s.d. **B.** Representative images of immunofluorescence in MDA-MB-231 treated as in A. **C.** Quantification of cells with complete YAP/TAZ nuclear exclusion. MDA-MB-231 cells were treated with vehicle (−), Zoledronic acid 30 μM (ZA) and Dasatinib 1 μM (D) alone or in combination (ZAD) for 24 h. Data are derived from *n* = 3 independent experiments where at least 300 cells were scored. Error bars represent mean ± s.d. **D.** Luciferase reporter assay (8XGTII–lux). Cells were transfected with control siRNA (siCTL) or with YAP/TAZ siRNA (siYT). After 24 h cells were treated with vehicle (−) or with statin 1 μM and Dasatinib 1 μM in combination (DS) for 24 h. Error bars represent mean ± s.d., from *n* = 3. **E.** Quantitative PCR (qPCR) analysis in MDA-MB-231. Cells were transfected with control siRNA (siCTL) or with YAP/TAZ siRNA (siYT). After 24 h cells were treated with vehicle (−) or with statin 1 μM and Dasatinib 1 μM in combination (DS) for 48 h. Error bars represent mean ± s.d., from *n* = 3 biological replicates. **F.** MDA-MB-231 and H1299 cells were treated with indicated compounds for 48 h. Representative blots are shown. **G.** Cell growth of MDA-MB-231 and H1299 cells treated with vehicle (−), statin 1 μM (S) and Dasatinib 1 μM (D) alone or in combination (SD) for three days. Error bars represent mean ± s.d., from *n* = 3 biological replicates. **H.** Cell viability of MDA-MB-231 cells treated with vehicle (−), Paclitaxel 1 μM (PTX) alone or in combination with Zoledronic acid 30 μM and Dasatinib 1 μM (PTX+ZAD) for 48 hour.

Statins are inhibitors of HMG-CoA-reductase, the limiting step enzyme of the metabolic mevalonate pathway and used in clinics for the treatment of hypercholesterolemia [34]. Several clinical and pre-clinical studies suggest that statins can exert an anti-tumour effect in particular biological contexts [35]. Importantly, we recently identified statins as inhibitors of YAP/TAZ activity in cancer cells [36]. Furthermore, YAP expression has been shown to be a predictive biomarker of Dasatinib response in breast cancer cells [37, 48]. Therefore when interrogated for drugs acting on cells with mutations in genes controlling the activity of YAP and TAZ the portal allowed the identification of molecules affecting YAP and TAZ function. We next wanted to test whether the pharmacological combination of statins and Dasatinib could have stronger effects in inhibiting YAP/TAZ nuclear localization and transcriptional activity. To this aim, we treated a panel of cancer cells from tissues in which the role of YAP and TAZ in tumorigenesis has been established (breast, lung, colon, prostate) [38] with statins and Dasatinib alone or in combination. Although both statins and Dasatinib, as single treatments, were effective in reducing YAP/TAZ nuclear localization (Supplementary Figure 2), the combination of these two drugs dramatically increased the number of cells showing a complete YAP/TAZ nuclear exclusion (Figure 4A and 4B). Similar results were obtained using an alternative mevalonate pathway inhibitor: zoledronic acid (ZA) (Figure 4C). Moreover, the concomitant treatment of MDA-MB-231 breast cancer cells with statins and Dasatinib completely blocked the YAP/TAZ transcriptional activity, as observed by the dramatic drop in the expression of the well established YAP/TAZ target genes CTGF, CYR61, ANKRD1 and BIRC5 and the TEAD-responsive reporter 8XGTII (Figure 4D and 4E) [36, 40]. Of note, the effect of Dasatinib and statins reduced YAP/TAZ target genes expression to the same extent obtained by YAP/TAZ siRNA transfection, suggesting that this combinatorial treatment can fully blunt the pro-oncogenic transcriptional activities of YAP/TAZ. Strikingly, in different cancer cell lines, statins and Dasatinib were significantly more active in inducing apoptosis (Figure 4F and 4G and Supplementary Figure 3A and 3B) when administered concomitantly.

YAP and TAZ are known to induce chemoresistance to taxanes in different cancer cells and their inhibition has been proposed as a strategy to sensitize cancers cells to standard chemotherapy [42]. Based on this, we hypothesized that combination of mevalonate pathway inhibitors and Dasatinib could efficiently sensitize cancer cells to sub-lethal doses of paclitaxel. As shown in Figure 4H, viability assay performed in MDA-MB-231 breast cancer cells with activated YAP/TAZ, confirmed that combination of Zoledronic acid and Dasatinib significantly sensitized cancer cells to paclitaxel treatment. These results demonstrate that combinations of Dasatinib and mevalonate pathway inhibitors could represent a pharmacological strategy to inhibit YAP/TAZ in cancer cells and to sensitize cells with active YAP/TAZ to standard chemotherapy.

## DISCUSSION

Targeted therapies directed against specific oncogenes are established strategies to successfully treat a growing number of cancers. Although many patients benefit of such therapeutic intervention, tumours often recur in few months due to acquired drug resistance, which calls for urgent development of novel strategies to identify drugs for personalized therapies [41].

Next generation sequencing of cancer cell lines, coupled with high throughput cell viability-based screening of small molecules, offers the possibility to rapidly identify many cancer-specific dependencies, potentially targetable with numerous chemical compounds. Nevertheless, the full exploitation of these “omics” databases is still hampered by gaps in the integration of genomics and pharmacological data. Here we developed MDP, an open access resource, which helps the researchers to unveil oncogene-induced dependencies by systematically coupling specific gene mutations with the pharmacological response to tens of thousands of compounds. MDP is available on-line and links genomic information from CCLE and NCI60 databases to pharmacological data from the NCI60 DTP screening allowing the identification of synthetic or natural compounds active in cancer cells with specific genetic features. The main goal of this web resource is to offer a very user-friendly platform for the execution of custom *in-silico* high-throughput screenings of thousand of compounds, with the possibility to querying associations in either direction (i.e., genes to drugs or drugs to genes) depending on the particular question of interest.

Since oncogenes may give rise to pharmacological dependencies only in particular tissues, MDP easily allows users to run the analysis selecting only cancer cells from a specific tissue thus leading to the identification of tissue-specific therapeutic biomarkers. Moreover the resource permits multiple mutation queries, enabling the identification of drugs active against cells with more than one mutated gene at the same time.

MDP, through the *from drug to gene* analysis, is capable of retrieving the most enriched mutations associated with sensitivity to the specific drug selected by the users and, in this way, might help researchers to identify potential drug-mutation dependencies in cancer cell lines, or unveiling new correlations between these mutated cancer cell lines and their sensitivity to a particular drug.

The reliability of this approach is guaranteed by the identification of already established and clinically proved gene-drug pairs, among them BRAF V600E mutation with clinically used BRAF/MEK inhibitors or EGFR mutations with EGFR inhibitors. Moreover, MDP suggests several novel dependencies, making it a powerful tool to guide the early phases of drug discovery or drug repositioning. As an example, querying MDP for drugs selectively active on cells bearing mutation in NF2 gene, an event that leads to the aberrant activation of the transcription cofactors YAP and TAZ, we found that the combinatorial use of two FDA-approved drugs, namely statins and Dasatinib, acts synergistically to strongly inhibit the activity of YAP and TAZ in different biological contexts.

YAP and TAZ are critical downstream effectors of actomyosin cytoskeleton acting as nuclear transducers of mechanical stimuli [40] and we and others, recently discovered that YAP and TAZ are activated by the metabolic mevalonate pathway through geranylgeranylation of RhoA, and their oncogenic activities can be efficiently blunted by using statins and bisphosphonates [36]. Furthermore, it has been recently shown that in cancer associated fibroblasts, downstream of actomyosin cytoskeleton and mechanical stress, the kinase Src is functionally required for YAP activation and its inhibition by Dasatinib can efficiently block YAP nuclear functions [49]. Therefore, the specific effect exerted by statins and Dasatinib in cells with YAP/TAZ hyperactivation could be ascribed to the combined inhibition of the signals that from the cytoskeleton converge on YAP/TAZ. YAP levels have been identified as a biomarker of Dasatinib sensitivity in breast cancer [48] and, in particular, basal-type triple negative breast cancer (TNBC) cells, which have highly activated Src and YAP, are more sensitive to Dasatinib treatment [46, 38].

The evidence that statin and Dasatinib, as demonstrated from our work, act synergistically to inhibit YAP/TAZ suggests that they could be used in combination and thus at lower concentrations, to inhibit YAP/TAZ activity and tumour growth in cancers characterised by hyperactivation of YAP/TAZ and, among them, in triple negative breast cancer and metastatic mesothelioma [42].

While the huge amount of compounds screened represents the strength of MDP, the limited number of cancer cells and genotypes used can depict a weakness. However, the NCI60 cell lines have been extensively used to identify novel predictive cancer biomarkers proving that the number of different genotypes is not necessarily a limitation [43–45]. Recent publications reported the generation of interactive resources useful to identify novel gene-drug dependencies [6, 7]. Although these databases are built from genomic characterization of more than 200 cell lines, thus showing higher genetic heterogeneity in comparison to MDP, the pharmacological data obtained by these resources are dramatically lower compared to our tool. Importantly, despite such differences, MDP and these other resources identified a number of similar gene-drug connections, such as the already established BRAF mutations with MEK inhibitors and EGFR mutations with tyrosine kinases inhibitors, as well as novel connections such as NF2 mutations with Dasatinib [6] and XIAP mutations with Brefeldin [7]. Thus, due to the very large number of pharmacological data deposited in its database, MDP can represent a reliable alternative to other predictor tools available on-line for the repositioning of several compounds for targeted therapy.

In conclusion, here we present MDP, an on-line tool able to perform prediction about cancer cell lines mutations and drugs sensitivity dependencies. MDP is, as far as we know, the largest database of pharmacogenomics structured to be a user-friendly tool for the investigation of drugs-mutations correlations, and can be queried either from the gene or drug perspective. Data obtained via MDP will help researchers in the challenge of patients' stratification, thus refining the parameters for personalized targeted therapies on the basis of their specific genetic abnormalities. We strongly believe that MDP provides a novel and comprehensive tool for the systematic identification of new biomarkers of drug sensitivity, setting the rationale for the design of new clinical trials and the discovery of novel anticancer drugs.

## MATERIALS AND METHODS

### Statistical analysis

Statistical analyses are based on the drug response data file GI_50_ Data (Sept 2014 release; https://wiki.nci.nih.gov/display/NCIDTPdata/NCI-60+Growth+Inhibition+Data), retrieved from the NCI60 DTP portal, and sequencing data and variant classifications retrieved from the CCLE (http://www.broadinstitute.org/ccle/data/browseData?conversationPropagation=begin) and NCI60 (http://discover.nci.nih.gov/cellminer/loadDownload.do) public repositories.

GI_50_ Data file contains a matrix of GI_50_ values for 50,816 molecules tested on 115 cancer cell lines. GI_50_ values are computed, for any compound, as minus the log_10_ of IC_50_, i.e., the drug concentration necessary to inhibit 50% growth of treated cells relative to untreated controls. Prior to analysis, for any single compound, first the GI_50_ is transformed back to IC_50_ and then the IC_50_ value is normalized dividing the IC_50_ of any cell line by the average of the IC_50_ across all 115 cell lines.

The normalized IC_50_ (in log_2_ scale) of a compound is used to define the response for any combination of drug and cell line in terms of i) *good response* if the normalized log_2_ IC_50_ is lower than two standard deviations of the distribution of all log_2_ IC_50_ in a given cell line, and ii) *bad response* otherwise.

In the *from gene to drug* analysis, given a specific set of mutation/s, compounds with increased activity in *cases* (cancer cell lines treated with the given compound and bearing the specific set of mutation/s selected by the user) as compared to *controls* (all the other cancer cell lines treated with the given compound), are identified ranking all compounds based on a score given by the fraction of *good responses* in *cases* multiplied by the fraction of *bad responses* in *controls*. This score ranks each drug based on the enrichment of *good responses* in the *case* group. The statistical significance (*p*-value) of this ranking is computed, for each drug, using a one-tailed Fisher's exact test for the enrichment of *good responses* in *cases* as compared to *bad responses* in *controls*, given the number of *bad responses* in *cases* and *good responses* in *controls*.

The same ranking function and statistical test is also used to identify the most enriched mutations starting from a drug when performing a *from drug to gene* analysis. In this case, the normalized log_2_IC_50_ of the selected compound is used to first retrieve the two groups of cell lines with *good* and *bad responses* for that drug. Then, for each mutation, we calculate the number of *cases*, i.e., the fraction of cell lines in the *good response* group bearing the considered mutation, and the number of *controls*, as the fraction of cell lines in the *bad response* group bearing the considered mutation. The score given by the fraction of *good responses* in *cases* multiplied by the fraction of *bad responses* in *controls* is again used to rank mutations and a Fisher's test for the enrichment of *good responses* in *cases* as compared to *bad responses* in *controls*, given *bad responses* in *cases* and *good responses* in *controls*, to assess significance.

### MDP web tool programming

MDP web interface has been written using PHP version 5.0, HTML 5.0 and CSS. Connections between pages and programming languages have been performed using Twig, a template engine that allows fast connections between PHP and HTML. Graphical representations in the results pages (i.e. barplots, pie charts, tables, and scatter plots) have been coded using JavaScript and jQuery, basing on the structure of the latest Google APIs release (2015). Statistical methods of analyses were performed using Python 2.7, through Pandas 0.16 module, and R 3.0. R software was used in the *from gene to drug* analysis, whereas Python 2.7 and Pandas were used to perform the *from drug to gene* statistical process. MDP is hosted on a Linux server with 512 GB RAM and 64 processors.

### Cancer cell lines/treatments

MDA-MB-231, HT29, PC-3, SKBR-3 and PANC-1 were cultured in DMEM supplemented with 10% FBS (fetal bovine serum) and antibiotics. H1299 cells were cultured in RPMI 1640 with 10% FBS and antibiotics. Cells have been authenticated by STR profiling and are free from mycoplasma contamination. The following compounds were purchased from Sigma Aldrich: statin (Cerivastatin, SML0005), Zoledronic Acid (SML0223), Paclitaxel (T7191). Dasatinib was purchased from Selleck (S1021). Cell lines used to build MDP have been already authenticated (Berretina et al. 2012, Lorenzi et al. 2009).

### YAP/TAZ nuclear localization

For the quantification of the number of cells with nuclear YAP/TAZ at least 300 cells from different fields were counted. Only cells with complete nuclear YAP/TAZ exclusion were scored as positive.

### Cell growth/viability assays

For cell growth assay cells (MDA-MB-231, H1299, HT-29, PANC-1) were plated in 96-well plates and treated as indicated. Cells were trypsinized, collected and counted at day 1, 2 and 3. Cell viability was assayed with ATPlite (Perkin Elmer) according to the manufacturer's instructions using the EnSpireMultilabel Reader (Perkin Elmer).

### Quantitative real-time PCR

Cells were collected in Qiazol lysis reagent (Qiagen) for total RNA extraction, and contaminant DNA was removed by DNase treatment. Retrotranscription was performed with the Quantitect reverse transcription kit (Qiagen). The obtained cDNA was properly diluted and used in qPCR reactions performed with SsoAdvanced SYBR Green Supermix (Biorad) using the CFX96 Touch Real-Time PCR Detection System and analyzed with Biorad CFX Manager software. Each experiment was performed at least three times Expression levels are always given relative to histone H3. The primers used were previously described [36].

### Transfections

siRNA transfections were performed with Lipofectamine RNAi-MAX (Life Technologies) in antibiotic-free medium according to the manufacturer's instructions. Negative control siRNA was: AllStars negative control siRNA Qiagen 1027281. siRNA sequences are: YAP (GACAUCUUCUGGUCAGAGA) and TAZ (ACGUUGACUUAGGAACUUU).

DNA transfections were done with Lipofectamine LTX and Plus Reagent (Invitrogen) or Lipofectamine 2000 (for H1299 cells; Life Technologies) according to the manufacturer's instructions.

### Luciferase assay

Luciferase assays were performed in MDA-MB-231 cells with the established YAP/TAZ-responsive reporter 8xGTII–lux. Cell lysates were analyzed using the Dual-Luciferase Reporter Assay System (Promega, cod. E1910). Luciferase reporter (300 ng cm−2) were transfected together with CMV–Renilla (30 ng cm−2) to normalize for transfection efficiency. Cells were collected 24 h after DNA transfection. For luciferase assays in siRNA-transfected cells, siRNA transfection was achieved first and, after 48 h, transfection of plasmid DNA was performed. Cells were collected 24 h after DNA transfection.

### Antibodies

The antibodies used for western blot and immunofluorescence were: anti-YAP/TAZ (1:1,000 for western blot, 1:100 for immunofluorescence; sc101199, Santa Cruz Biotechnology), anti-actin (1:2,000; C11, Sigma), anti-Cleaved Caspase 3 (1:1000; D175, Cell Signaling).

### Immunofluorescence and western blot

Immunofluorescence staining was performed as previously described [36]. Briefly, cells were fixed in 4% paraformaldehyde for 10 min, washed in PBS, permeabilized with Triton 0.1% for 10 min and blocked in PBS FBS 3% for 30 min. Antigen recognition was done by incubating primary antibody for 1 h at 37°C and with Goat anti-mouse Alexa Fluor 568 (Life Technologies) as secondary antibody for 30 min at 37°C. Nuclei were counterstained with Hoechst 33342 (Life Technologies). Western blot analysis was performed as previously described [36].

## SUPPLEMENTARY FIGURES


