# Supplementary material for: MDP, a database linking drug response data to genomic information, identifies dasatinib and statins as a combinatorial strategy to inhibit YAP/TAZ in cancer cells
Source: Oncotarget. 2015 Oct 15;6(36):38854–65. doi: 10.18632/oncotarget.5749 (PMC4770742; doi:10.18632/oncotarget.5749)
Supplement: Supplementary file 1 [file oncotarget-06-38854-s001.pdf]

SUPPLEMENTARY FIGURES

Enriched Drug Family

DRUG FAMILY	DRUGS IN TOTAL	DRUGS WITH SCORE > 0.3	DRUGS WITH SCORE > 0.6	P-VALUE OF DRUGS WITH SCORE > 0.3	P-VALUE OF DRUGS WITH SCORE > 0.6
Statin	6	6	2	0	0.00018
Imatinib analog	24	5	2	0.00098	0.00324
Quinone/Quinoline	1220	67	6	4.0E-5	0.26127
Sulfonate	113	12	1	0.00037	0.32914
Others	49225	1505	168	1	0.9889
Nitrosureas cross-linking agents	8	1	0	0.23534	1
Platinum complexes	98	1	0	0.96274	1
Anthracyclines	33	3	0	0.0941	1
Purine/Pirimidine analogues	20	2	0	0.13987	1
Specific target inhibitors	13	2	0	0.06665	1
Vinca alkaloides	13	1	0	0.35341	1

Supplementary Figure S1: NF2 enrichment analysis. The table lists the different drug families statistically significant on all CCLE cancer cell lines bearing a missense or a non-sense mutation in the NF2 gene. Using one-tailed Fisher's test (p-value < 0.05), statins and Imatinib analogues resulted the most enriched drug families with selective activity.

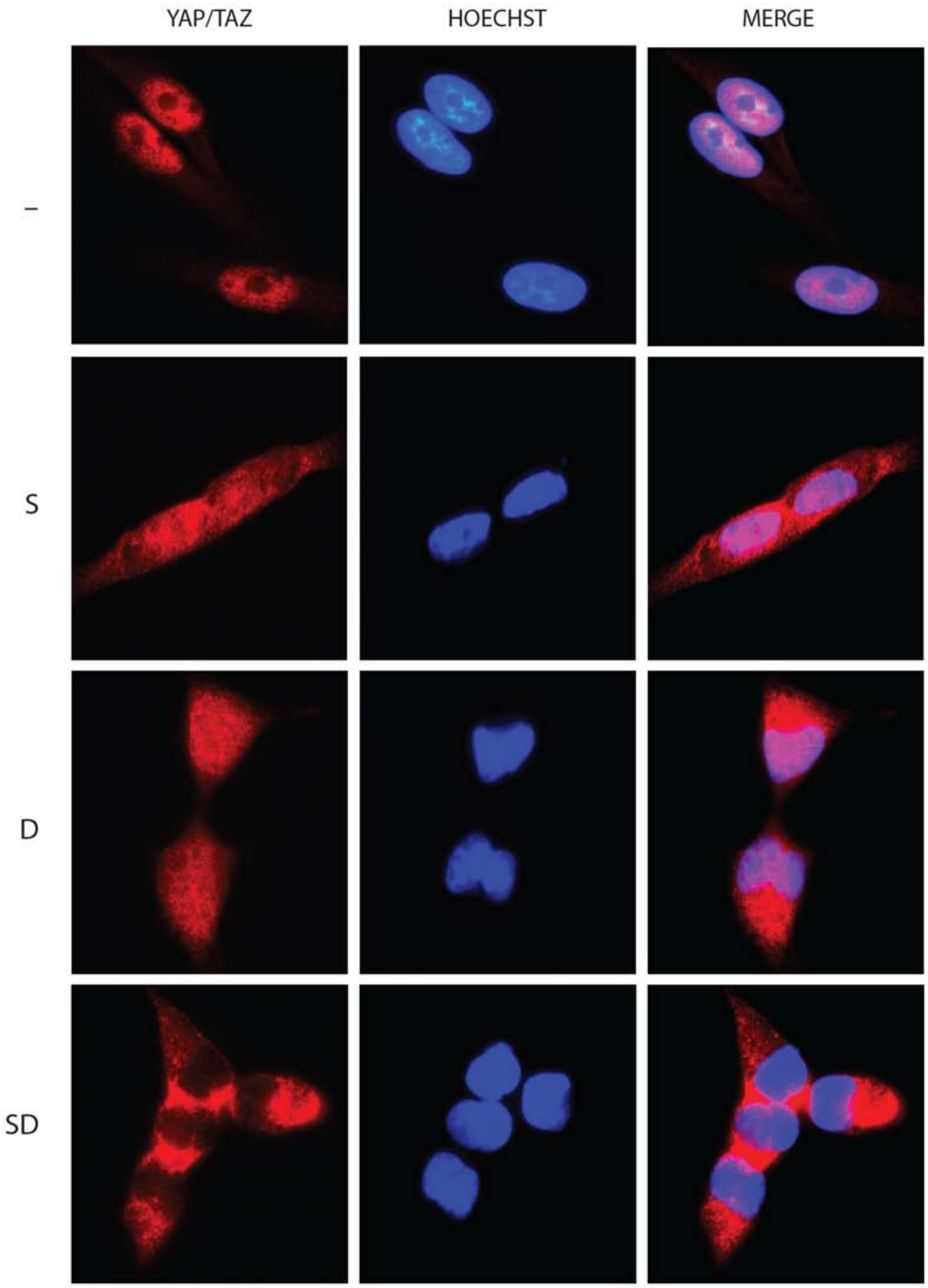

Supplementary Figure S2: Dasatinib and statin induce YAP/TAZ cytoplasmic retention. MDA-MB-231 cells treated with vehicle (-), statin 1 μM (S) and Dasatinib 1 μM (D) alone or in combination (SD) for 24 h. Representative images of immunofluorescence are shown.

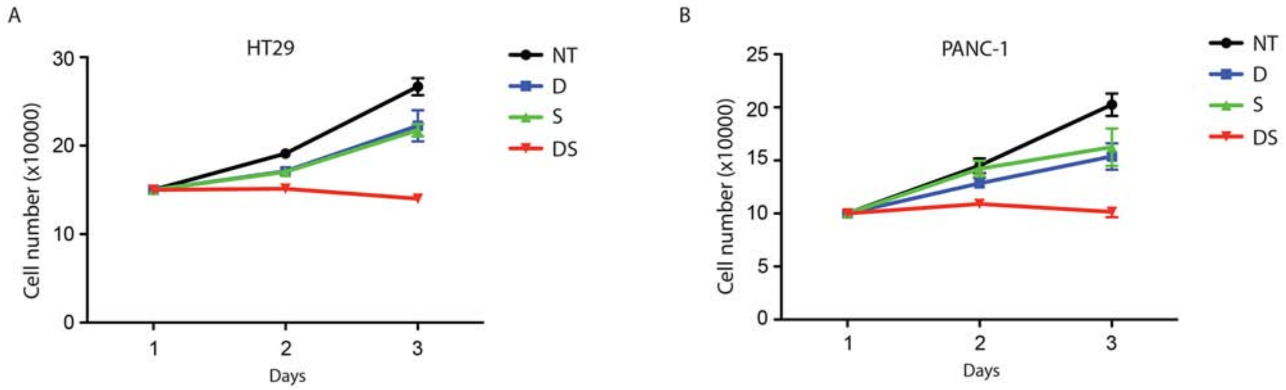

Supplementary Figure S3: Dasatinib and statin combination inhibits cell growth. Cell growth of HT29 **A.** and PANC-1 **B.** cells treated with vehicle (–), statin 1 µM (S) and Dasatinib 1 µM (D) alone or in combination (SD) for three days. Error bars represent mean ± s.d., from n = 3 biological replicates
